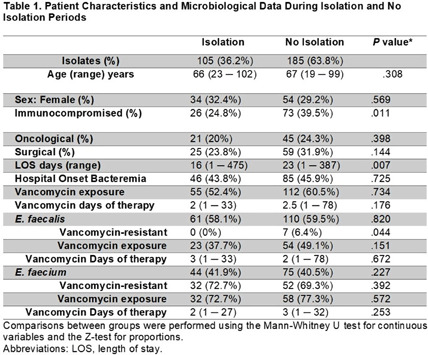# Impact of Stopping Contact Isolation for Vancomycin-Resistant Enterococci on vanA Plasmid Transmission, Northern California, 2021–2023

**DOI:** 10.1017/ash.2025.230

**Published:** 2025-09-24

**Authors:** Guillermo Rodriguez Nava, Matt Grieshop, Alessandro Zulli, Eugenia Miranti, Wajeeha Tariq, Erika Paola Viana Cardenas, Mindy Sampson, Alexandria Boehm, Ami Bhatt, Jorge Salinas

**Affiliations:** 1 Stanford University School of Medicine; 2 Stanford University; 3 Stanford Health Care

## Abstract

**Introduction:** Enterococci are the third most common healthcare-associated pathogen, with 30% of isolates resistant to vancomycin (VRE). Resistance is often conferred by the vanA gene cluster on transposon Tn1546 and is frequently plasmid-borne. The suspected role of person-to-person transmission prompted the recommendation for VRE isolation precautions in 1995. However, quasi-experimental studies in hospitals discontinuing these precautions found no significant increases in VRE infections or bacterial clone spread using short-read whole genome sequencing (WGS). We used long-read WGS to analyze vanA plasmid transmission dynamics after discontinuing isolation precautions for VRE at Stanford University Hospital. **Methods:** This study was conducted at Stanford University Hospital, an 800-bed quaternary referral and transplant center. Routine contact precautions for VRE were discontinued on October 1, 2021. Blood culture Enterococcus faecalis and E. faecium isolates collected during 2021 (prior to and following discontinuation) were included, along with additional isolates retrieved from January–October 2023. Bacterial whole genome sequencing with long-read nanopore technology was performed. Custom analyses were performed on the assembled genomes. Patient data were collected retrospectively. **Results:** We retrieved 105 blood culture isolates (36.2%) from the isolation period (January–October 2021) and 185 isolates (63.8%) from the no isolation period (October–December 2021, January–October 2023), representing 202 unique patients. Patient characteristics and microbiological findings are shown in Table 1. Only 4.3% (7/171) of E. faecalis and 70.5% (84/119) of E. faecium isolates were vancomycin-resistant. Long-read WGS revealed no clustering between the isolation and no isolation periods. (Figure 1A and B); however, a dominant E. faecium ST117 cluster was seen, while E. faecalis showed greater diversity (Figure 1C). There were only four pairs of putative transmissions **Conclusion:** The discontinuation of contact isolation precautions at Stanford Hospital did not result in an increase in genetically related Enterococci or genetically related vanA plasmids among patients with Enterococcal bacteremia.

**Figure:**
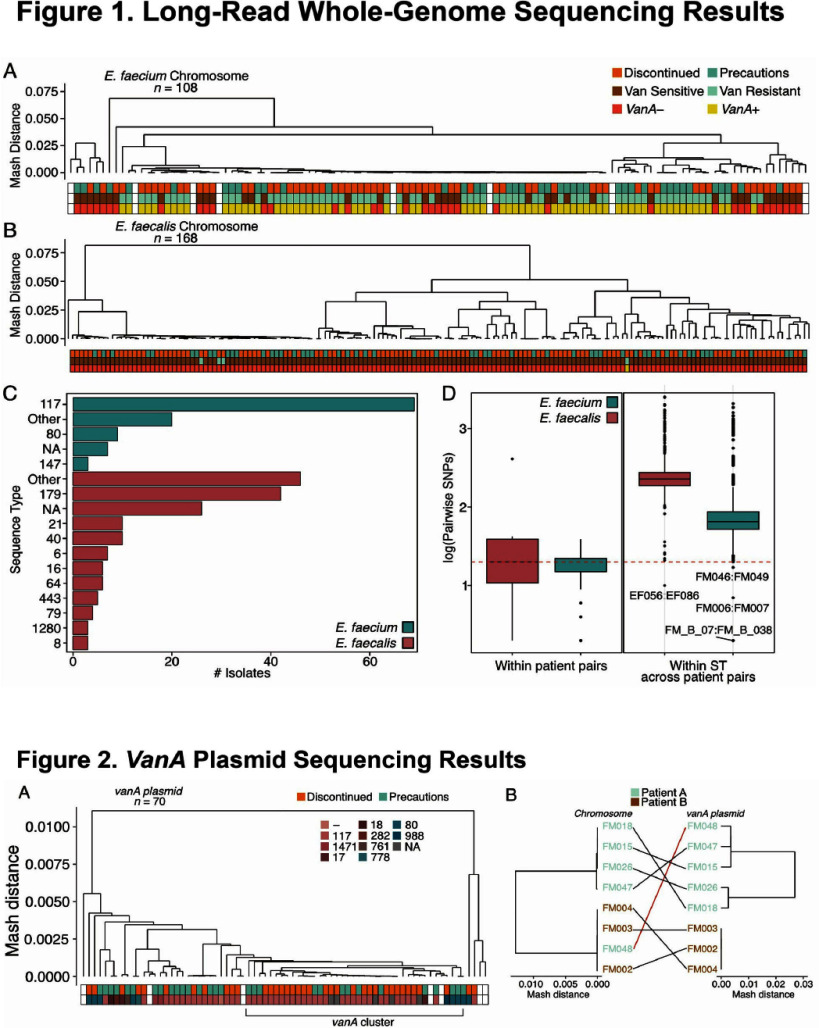

**Figure:**